# Investigations for Material Tracing in Selective Laser Sintering: Part Ι: Methodical Selection of a Suitable Marking Agent

**DOI:** 10.3390/ma16031043

**Published:** 2023-01-24

**Authors:** Tom Eggers, Frank von Lacroix, Fridolin van de Kraan, Ann-Kathrin Reichler, André Hürkamp, Klaus Dröder

**Affiliations:** 1 Volkswagen AG Wolfsburg, Berliner Ring 2, 38438 Wolfsburg, Germany; 2 Institute of Machine Tools and Production Technology, Technische Universität Braunschweig, Langer Kamp 19b, 38106 Braunschweig, Germany

**Keywords:** additive manufacturing, selective laser sintering, tracing, polymers, smart materials, modified polymers, process optimization, process predictability, recycling, circular economy

## Abstract

Selective laser sintering (SLS) with polymers is currently at the transition stage for the production of functional components and holds great potential to revolutionize conventional production processes. Nevertheless, its application capability is confronted by newly imposed requirements regarding reliability and reproducibility. To safeguard these requirements, a deeper process understanding of material aging mechanisms in polymeric materials is needed. In order to enable the traceability of the materials as well as the identification of defective components with subsequent tracing of the cause, the use of a material marking process represents an alternative. SLS in combination with material marking is proving to be an efficient option for reproducible, high-quality manufacturing based on an increased understanding of the process. In this study, the idea of a marker-based traceability methodology for the purpose of process optimization is presented. Fundamental to the subsequent experimental investigation of the marking agent suitability, this work first focuses on the systematic selection of a suitable marking agent for use in SLS. Based on an analysis of the sinter material to be marked and a set of marking technologies, as well as using the selection methodology, the modified polymer marking technology was evaluated as the most suitable marking technology.

## 1. Introduction

Selective laser sintering (SLS) with polymeric materials represents the additive manufacturing process that is currently in transition between its application in prototype development and full manufacturing technology for functional components. In addition to the suitability of the process for future industrial applications, the process offers the potential integration into volume production. SLS is a powder-based process in which components are generated through a layer-by-layer deposition and selective solidification of primarily polymeric materials using heat input [1,2,3,4,5,6]. Taking into account minimal distances between the components, the building chamber can be fully utilized, and no support structures are required due to the surrounding powder. The remaining unsolidified powder can be predominantly reused [3,5,6,7,8]. A significant restriction of the process is that the used powder material is undergoing a spectrum of material aging processes [3,9,10,11]. The various aging mechanisms of importance in the SLS process include molar mass increase due to solid phase post-condensation [12,13,14,15], thermo-oxidative material degradation [10,16], hydrolytic depolymerization [10,17], crystallinity change [15], and additive degradation [2,18]. Whereas the aging phenomena of polymers are often only visible macroscopically at an advanced stage of aging, the changes that have already taken place in the polymer lead to altered material characteristics that are difficult for the user to identify [10]. Since the influence of the specific material properties has an impact on the component quality and the process stability of the procedure [2,3,9,10,14,16,19,20], the industrial application of SLS is limited. Up to now, these aging phenomena have been compensated by recycling strategies, in order to guarantee consistent component quality and ensure process stability. With regard to polyamide 12, it is common practice to prophylactically refresh powder that is already used in the process with 30% to 50% new powder based on empirical values [2,3,5,6,9,16,19,20,21]. In this context, the heterogeneous properties of a powder mixture of new and recycled powder represent a significant cause of variation [9,22].

There are studies focusing on the determination of material quality limits or an evaluation system to predict the time of occurrence of material aging effects [15,21,23,24,25], as well as the compensation of aging effects in polymers by adapted process control [2,25,26]. However, according to Schmid [2] and Mielicki [22], it is not yet possible to control the powder mixture being processed with regards to the various powder constituents and their conditions. Previous strategies [2,9,16,19,20] for understanding the aging effects of materials and the composition of the powder mixture and for setting spread-reducing process parameters are not effective because they are logistically and planning-wise complex and prone to error [9,22]. At present, there is no agile system for fast and simple detection of the aging state of an SLS polymer powder, which enables timely correction of the process parameters for compensation. Hence, this lack of information ultimately manifests itself in the finished product in the form of surface defects, geometric deviations, or an unacceptable property profile [2,22]. Quality assurance measures must focus in particular on the reduction and understanding of variations in material properties [22].

A promising research approach is to increase the information density in materials and components as a result of material marking, which follows the requirements for future manufacturing processes mentioned by Esper [27]. Material and component marking processes are already established technology standards in a number of application areas [28]. While tracking refers to the downstream traceability in the process flow, tracing describes the upstream traceability [28,29,30]. This includes the origin of a unit, starting with the end product and extending to the starting materials, components, and treatment parameters used [31]. In order to enable tracing of the polymer materials used in SLS as well as the identification of defective components with subsequent tracing of the cause, the application of a material marking process represents an alternative method. The tracing approach enables continuous, material inherent information storage about powder and component phases during SLS. A definition of this tracing approach for an investigation in the context of SLS is not yet available, but results as follows, taking the Global Tracibilty Standard 2.0 [28] into account: The tracing of materials and components on the basis of defined analyzation points within the process chain of SLS, as well as the thereby complete information continuity of the stored data.

In the sense of leap frogging, individual stages of the previous development process of recycling strategies are omitted and skipped by the tracing approach. If the material history or composition is known, conclusions can be drawn about the material quality. The provision, processing, and intelligent use of valid process, plant, and component data for the purpose of in-process quality assurance increase the significance of SLS in an industrial context. Material traceability in the SLS process is achieved by the stepwise addition of one or more marking agents at different stations of the SLS process or with each addition of new powder and the readout of the marking agents at defined analyzation points. It is envisaged that the material composition is determined either by qualitative or quantitative analysis of the marking agents, as well as a combination of both variants. The use of different marking technologies is also conceivable. Depending on the used marking agent, the tracing strategy has to be designed. If variations of the material properties are detected within the manufacturing process, the process parameters can be specifically adjusted to reduce the influence of varying material properties [9,22,32]. For this purpose, robust parameter sets with suitable limit values for the material quality with respect to deviating material properties are already being developed [23,24]. Fixed sets of process parameters can be selected or interpolation can be performed between individual stages. By gradually refreshing the material with new powder, the proportions of the older marking agents become smaller and smaller, as does the respective proportion of aged powder. Thus, not all marking agents have to be present in the same concentration. Below a certain concentration, the marking falls below the detection limit and is, therefore, not considered further. The principle of tracer-based process optimization through material marking thus provides the basis for a continuous improvement process of the production and, ultimately, of the product. This enables software-based product optimization and adaption of the production system to the respective circumstances [33,34,35,36].

SLS in combination with material marking has not been explored to date, but is potentially proving to be an efficient way to manufacture reproducibly and with high quality, based on an information-dense material [37]. The first mention of the addition of a so-called “marking agent” to powders is found in Fornos et al. [38]. For the implementation and application of a marking technology applicable to SLS, a high degree of maturity is required at this very time [39,40,41,42]. Fundamentally for the subsequent experimental investigation of the suitability of the marking agents in part II of the study, the aim of this work is initially the systematic selection of a suitable marker for use in SLS, taking into account the sinter material to be marked. Therefore, a set of marking technologies is evaluated using the applied selection methodology. The selection process is based on the available information on the individual marking technologies. The testing of the marking agents to validate the selection result is part of a following investigation. The methodology for selecting a marking agent for SLS is based on a chronological combination of individual tasks of the utility analysis and the PROMETHEE method. Thus, the methodology is a multi-criteria decision support tool according to the Multi-Attribute Decision-Making method [43,44,45,46,47]. Compared to previous methods, the new methodology offers a systematic approach with a clearly definable set of alternatives and easier, transparent implementability. A numerical index offers the user the advantage of a simple evaluation of the result. Depending on the selected marking agent and its operating principle and properties, a suitable tracing strategy will be developed in part II of the study.

## 2. Materials and Methods

### 2.1. Sinter Material

In this work, the powdered polyamide 12 sinter material LUVOSINT PA12 9270 BK from the manufacturer Lehmann&Voss&Co. KG (Hamburg, Germany) is used. According to the information provided by the manufacturer [48], the material has a specific gravity of 1.02 g/cm^3^. Due to the necessity of a sinter window for the successful process flow in SLS, only semi-crystalline thermoplastics are processed. The choice of material is justified by the fact that polyamide 12 is the most commonly used material in SLS [2,3,49,50].

### 2.2. Marking Materials

In this work, three different marking agents are investigated with regard to their suitability for material marking in SLS using the sinter material employed. Possibilities include modified polymers, fluorescent particles, and silicate-encapsulated deoxyribonucleic acid (DNA). The possibility of modified polymers comes from the POLTAG^®^ technology from the company Polysecure GmbH (Freiburg, Germany). The fluorescent particles are available as inorganic, red fluorescent pigments and are purchased from the company Tailorlux GmbH under the brand name Tailor-SAFE^®^. The information available on marking materials at the time of processing serves as the data basis for the investigation. Manufacturer-specific information on the materials as well as information available in the literature is used (Table 1). The silicate-encapsulated DNA is currently not commercially available, so the information available in the literature is used in this respect.

### 2.3. Particle Analysis

For the analysis of the particle shape and particle size distribution (PSD) through a dynamic image analysis according to ISO 13322-2:2021-12 [66], the particle analyzer type Camsizer XT from Retsch Technology GmbH (Haan, Germany) is used. For the analysis of the particles, measurements with five million recorded particles are carried out in each case. As a result of the measurements, the sphericity and aspect ratio are available as mean values. In addition, the D10, D50, and D90 values are output. This measurement does not provide for a standard deviation. Regarding particle shape, the focus is on sphericity and aspect ratio, whose maximum value is 1 [2,67].

### 2.4. Scanning Electron Microscope

The scanning electron microscopic (SEM) images of the investigated materials are recorded with a Tescan Mira 3 SEM system (Dortmund, Germany) from the manufacturer Tescan GmbH. The microscope is operated with an accelerating voltage of 15 kV and detection is performed via secondary electrons. The samples are first sputtered with gold for 40 s so that the particle surface becomes electrically conductive [68].

### 2.5. Differential Scanning Calometry Testing

Differential Scanning Calometry (DSC) is performed with the Mettler Toledo DSC-822 measurement system (Gießen, Germany) from Mettler Toledo GmbH. The measurement is performed under nitrogen atmosphere. A sample weight of 10 mg ± 2 mg is used for the measurement. Heating and cooling cycles between 25 °C and 230 °C are performed at a rate of 10 °C/min. The measurement is carried out according to DIN EN ISO 11357-1:2017-02 [69]. The DSC analysis is used only to determine the melting temperature of the sinter material. The measurement does not provide for a standard deviation.

### 2.6. Material Properties

The previously mentioned methods is used to investigate the sinter material. Table 2 lists the investigated material properties for the used sinter material.

**Figure 1:**
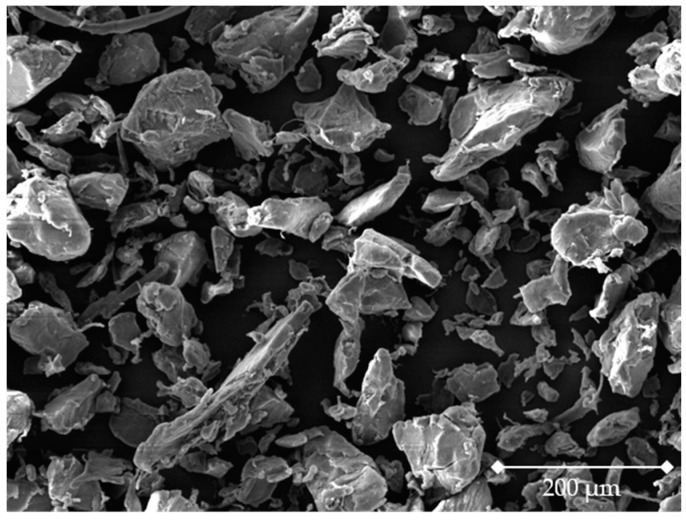
SEM image of the used sinter material. Produced with Tescan Mira 3 at 150× magnification.

Figure 1 shows the SEM image of the sinter material. A jagged particle shape is present. This appearance is confirmed by the sphericity and aspect ratio of the material (Table 2). The color expression of the sinter material is black.

### 2.7. Methodology for the Systematic Selection of Marking Materials

**Figure 2:**
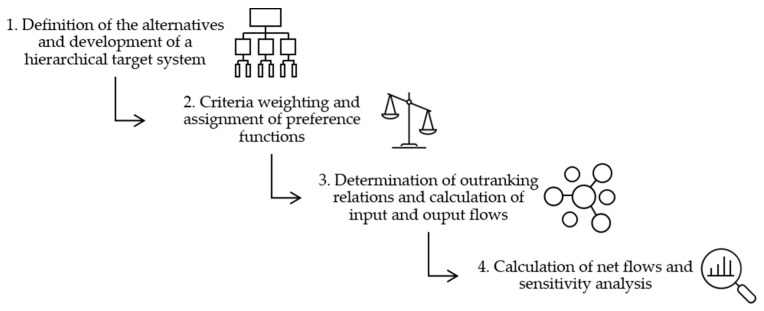
Establishment of the methodology for the systematic selection of a marking agent for a material marking in SLS.

The decision support process within the method for systematic selection of a marking agent for use in SLS is divided into four main phases (Figure 2). The methodology is based on the structure of a hierarchical target system according to the utility value analysis. The target system is then used for an evaluation of the alternatives according to the PROMETHEE method. The target system ensures a structured procedure for their derivation. The analysis by means of PROMETHEE II then enables a clear structure of the decision and a presentation of the results as a total order by generating a ranking list.

#### 2.7.1. Definition of the Alternatives and Development of a Hierarchical Target System

According to the value system at hand, the different alternatives are defined by the deciding person or group of persons. This contains all options that represent a possible result of the decision. As shown in Table 1, three marking technologies are investigated for their suitability as marking agents for SLS:

- Alternative a_1_: Modified polymers;
- Alternative a_2_: Fluorescent particles;
- Alternative a_3_: Silicate-encapsulated DNA.

Then, according to the first subtask of the utility value analysis [44], a hierarchical target system has to be developed, whereby the superordinate main target is divided into resulting sub-targets following the “top-down” design. The “top-down” methodology offers the advantage of being fully aware of the superordinate main goal [45]. Accordingly, the selection of the most suitable marking agent for SLS is identified as the overriding main objective. With regard to the requirements for a marking agent, it is basically assumed that influence on the requirements for a material for SLS mentioned in Schmid [2] also apply to a marking agent for SLS. In addition, Balzereit [70] sets out requirements for an additive for SLS, which are also taken into account in the definition of requirements. Based on the requirements from the literature, the process principle of SLS, and the available state of information on the individual material marking technologies, the following two main target groups are defined:

- Objective main group 1: Simple application and evaluation;
- Objective main group 2: Best possible industrial suitability.

The respective goals belonging to the target main groups are defined in the following. There may be overlap areas between individual decision dimensions, which can be merged to form combined requirements.

Target main group 1 includes the coloring of the marking agent and the introduction of the marking agent into the sinter material, the analysis effort, and the type of testing. It is considered whether an additional process step has to be implemented in the SLS process chain to ensure a homogeneous introduction of the marking agent into the sinter material [71,72,73]. During the introduction of the marking agent to the SLS, the influencing factor of powder rheology is particularly decisive as an evaluation criterion [2]. The powder rheological properties result from the particle shape and PSD [2,3]. As a general rule, the flow behavior of the powdered sinter material must not be influenced by the marking agent [2,5,18,74,75,76,77,78,79]. With regard to the particle shape, a shape that is as spherical as possible or analogous to the sinter material (Table 2 and Figure 1) is preferred [2,74,80,81]. Furthermore, the PSD of the marking agent should be in the range of the distribution of the sinter material (Table 2) [2,74,80,82]. In addition, for applicability as a marking technology, the marking agent should ostensibly not affect the aesthetics and perception of the sinter material and generated product [2,83,84]. In terms of aesthetics, the target group of optics is defined as a customer-relevant feature. Among other things, the component’s appearance is influenced by the color of the component, so marking agents that do not cause a color change in the sinter material (Figure 1) are preferred [2,83,84]. Moreover, a higher workload is disadvantageous compared to marking systems whose insertion can be carried out inhomogeneously, as they can already be integrated into the existing process steps of SLS. The analysis effort is described in terms of the time required to evaluate a material sample. Destructive testing is to be avoided with regard to the recycling of powder and the damage to components. Thus, a process without destruction of the powder or component is evaluated as positive.

The industrial suitability of the marking agent, as regraded by target main group 2, is defined by the harmlessness, commercial availability, possible code number, and resistance of the marking agent. Unless the marking agent is proven to be harmless via safety records, it is not suitable for a material marking technology [72]. Commercial availability describes the possibility of acquiring a marking agent in a suitable form from a supplier. The independent development and production of a marking agent is not preferred in the context of this work. The information content of a marking agent is described by the number of unique codes that can be generated. The number of codes is a measure for the application spectrum of the respective marking agent. The process temperature of SLS also requires the marking agent to be thermally stable. The marking agent must be detectable both in the recycled powder and in the component, and there must be no influence on the printing process due to the decomposition of the marking agent [2,3,6,35,71,72]. The melting temperature of the used sinter material (Table 2) is taken as the reference point for assessing the thermal resistance of the marking agent. Since SLS involves temperatures up to 210 °C [6], a safety margin of 10% is considered. A higher thermal resistance beyond the minimum requirement does not represent any added value for the application in SLS.

**Figure 3:**
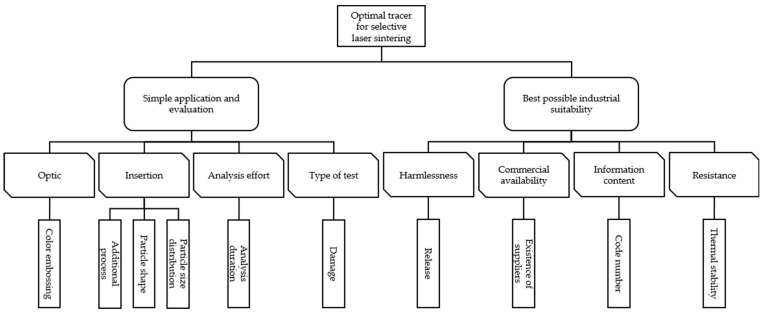
Established hierarchical target system for the present use case.

Based on the described targets, the hierarchical target system is set up (Figure 3).

Based on the hierarchical target system (Figure 3) and taking into account the used sinter material, the evaluation points of the individual criteria are obtained (Table 3).

#### 2.7.2. Criteria Weighting and Assignment of Preference Functions

After defining the target system (Figure 3), specific weights are assigned to individual criteria (Table 3). The weighting of the criteria is based on a pairwise comparison by the authors. The following point scale is chosen to represent the preferences. Here, A is the criterion under consideration (row) and B is the comparison criterion (column). The even intermediate values allow a finer distinction and are used accordingly in the evaluation:

- 1: strong preference of B over A;
- 3: equivalence of A and B;
- 5: strong preference of A over B.

The ratio of the sum of the single criterion to the total sum of the criteria is formed. Table 4 shows that the criterion of harmlessness (C7) was weighted as the most important criterion and the criterion of color expression (C1) as the least important criterion.

Thereupon, for each individual criterion, a preference function has to be defined additionally, on the basis of which the different alternatives are evaluated with respect to the criterion to be examined. The preference functions indicate the degree of preference of the user of an alternative a_n_ over an alternative a_m_ as a value within the interval from zero inclusive to one inclusive. Accordingly, the value zero expresses indifference and one expresses strong preference of a_n_ over a_m_ Equation (1) [43].

(1) $$P\left(a_{n}{,\;a}_{m}\right)=\{\begin{array}{cc} 0 & ,\;\mathrm{if}\;f\left(a_{n}\right)\leq f\left(a_{m}\right)\\ p\left[f\left(a_{n}\right),f\left(a_{m}\right)\right] & ,\;\mathrm{if}\;f\left(a_{n}\right)\text{>}f\left(a_{m}\right) \end{array}$$

$$\begin{array}{cc} P\left(a_{n}{,a}_{m}\right)\left[-\right] & {\mathrm{Preference}\;\mathrm{function}\;\mathrm{from}\;a}_{n}{\;\mathrm{to}\;a}_{m}\\ p\left[f\left(a_{n}\right),f\left(a_{m}\right)\right]\left[-\right] & {\mathrm{Degree}\;\mathrm{of}\;\mathrm{preference}\;\mathrm{from}\;a}_{n}{\;\mathrm{to}\;a}_{m}\\ f\left(a_{n}{,a}_{m}\right) & {\mathrm{Criterion},\;\mathrm{applied}\;\mathrm{to}\;a}_{n}{\;\mathrm{and}\;a}_{m} \end{array}$$

The assignment of used preference functions is shown in Table 5 and is based on the judgment points defined in Table 3. Depending on the preference function, a decision can be made between indifference, strict preference, and an expression of weak preference [43,45]. Further information on the preference functions used in this work is listed in Brans and Vincke [43] and Geldermann and Lerche [45].

#### 2.7.3. Determination of Outranking Relations and Calculation of Input and Output Flows

Once the criterion weights of the individual criteria (Table 4) and preference functions (Table 5) have been determined, the outranking relations of the alternatives are calculated on the basis of these. First, the preference values of the individual alternatives are determined on the basis of the selected preference functions. The product of the preference value of an alternative and the respective criteria weight results in a partial term for the calculation of the outranking relations. The sum of the partial terms results in the outranking relation of the examined alternative Equation (2) [43,45,47].

(2) $$\pi \left(a_{n}{,a}_{m}\right)=\overset{n}{\underset{k=1}{\sum }}w_{k}\cdot p_{k}\left(a_{n}{,a}_{m}\right)$$

$$\begin{array}{cc} \pi \left(a_{n}{,a}_{m}\right)\left[-\right] & {\mathrm{Outranking}\;\mathrm{relations}\;\mathrm{from}\;a}_{n}{\;\mathrm{to}\;a}_{m}\\ w_{k}\left[-\right] & \mathrm{Criterion}\;\mathrm{weight}\\ p_{k}\left(a_{n}{,a}_{m}\right)\left[-\right] & {\mathrm{Preference}\;\mathrm{value}\;\mathrm{of}\;\mathrm{the}\;\mathrm{alternative}\;a}_{n}{\;\mathrm{over}\;a}_{m} \end{array}$$

Following the calculation of the outranking relations of all available alternatives, the input and output flows are calculated. The output flow of an alternative results from the normalized sum of the respective outranking relations Equation (3) [43,45,47]. Here, the summed results indicate in each case to what extent an alternative a_n_ is preferred over an alternative a_m_. The input flow of an alternative is, therefore, calculated from the normalized sum of the respective outranking relations Equation (4) [43,45,47]. These provide information on the extent to which the alternative under consideration is inferior to the other alternatives [43,45,47].

(3) $$\phi_{n}^{+}=\frac{1}{i-1}\cdot \overset{i}{\underset{k=1}{\sum }}\pi \left(a_{n}{,a}_{m}\right)$$

$$\begin{array}{cc} \phi_{n}^{+}\left[-\right] & \mathrm{Output}\;\mathrm{flow}\;\mathrm{of}\;\mathrm{an}\;\mathrm{alternative}\;n\\ \pi \left(a_{n}{,a}_{m}\right)\left[-\right] & {\mathrm{Outranking}\;\mathrm{relation}\;\mathrm{from}\;a}_{n}{\;\mathrm{to}\;a}_{m} \end{array}$$

(4) $$\phi_{n}^{-}=\frac{1}{i-1}\cdot \overset{i}{\underset{k=1}{\sum }}\pi \left(a_{m}{,a}_{n}\right)$$

$$\begin{array}{cc} \phi_{n}^{-}\left[-\right] & \mathrm{Input}\;\mathrm{flow}\;\mathrm{of}\;\mathrm{an}\;\mathrm{alternative}\;n\\ \pi \left(a_{m}{,a}_{n}\right)\left[-\right] & {\mathrm{Outranking}\;\mathrm{relation}\;\mathrm{from}\;a}_{m}{\;\mathrm{to}\;a}_{n} \end{array}$$

The resulting matrix with outranking relations and input and output flows is shown in Table 6.

#### 2.7.4. Calculation of Net Flow and Sensitivity Analysis

For evaluation, the previously calculated input and output flows (Table 6) are displayed graphically. The net flow is formed from the input and output flows for evaluation according to PROMETHEE II Equation (5) [43,45,47].

(5) $$\phi_{n}^{\mathrm{net}}=\phi_{n}^{+}\;-\;\phi_{n}^{-}$$

$$\begin{array}{cc} \phi_{n}^{\mathrm{net}}\left[-\right] & \mathrm{Net}\;\mathrm{flow}\;\mathrm{of}\;\mathrm{an}\;\mathrm{alternative}\;n\\ \phi_{n}^{+}\left[-\right] & \mathrm{Output}\;\mathrm{flow}\;\mathrm{of}\;\mathrm{an}\;\mathrm{alternative}\;n\\ \phi_{n}^{-}\left[-\right] & \mathrm{Input}\;\mathrm{flow}\;\mathrm{of}\;\mathrm{an}\;\mathrm{alternative}\;n \end{array}$$

The results from the net flow calculation are to be ordered from largest to smallest net flow. The resulting complete preorder represents the alternative with largest net flow, as preferred, with respect to the evaluation [43,45,47]. Subsequently, the result is subjected to a sensitivity analysis to control the influence of individual weighting factors (Table 4) and the choice of preference functions (Table 5) [45]. An examination of the criteria weighting is performed by means of pairwise comparison with the deletion of the worst and best rated criterion. If a change in the ranking of the remaining criteria, a so-called “rank reversal”, occurs during a new evaluation, it indicates an instability within the evaluation system [45]. If the number of criteria available is insufficient for a rank reversal investigation or if a more far-reaching analysis is required, a variation in the criteria weights can provide an assessment of the sensitivity of the system.

## 3. Results

The various steps of the described methodology (Figure 2) are applied to the present use case and the results are presented.

### 3.1. Determination of the Outranking Relations and Calculation of the Input and Output Flows

The preference values are determined on the basis of the respective preference functions for all alternative combinations considered. Then, using Equation (2), the outranking relations of all alternative combinations are determined and transformed into Table 7. Based on Equations (3) and (4), the out- and input flows are then calculated.

### 3.2. Calculation of Net Flows for Evaluation According to PROMETHEE II

**Figure 4:**
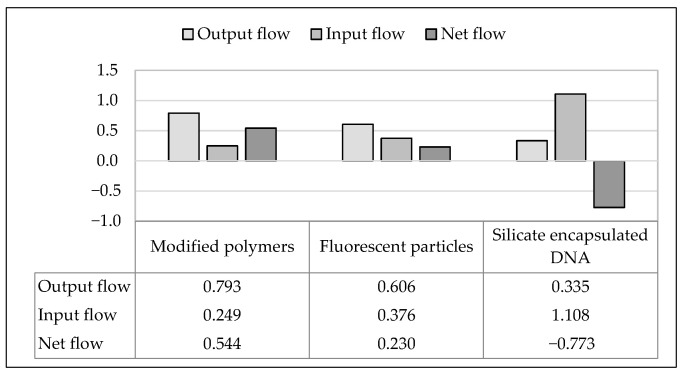
Presentation of results for the examined alternatives.

Based on the output and input flows of the alternatives listed in Table 7, the net flows are calculated using Equation (5) and shown in Figure 4.

The resulting total order of the studied alternatives is presented in the form of a ranking list in Table 8.

According to the evaluation, the modified polymers (a_1_) are the most favorable for the present application, followed by the fluorescent particles (a_2_).

### 3.3. Sensitivity Analysis

The robustness of the obtained result is checked by a sensitivity analysis using the “rank reversal” approach [45] when comparing the criteria weights pairwise. The criteria C7 as the most important and C1 as the least important are deleted (Table 4). As a result, a new Table 9 is obtained. The weighting and placement of the remaining criteria in relation to each other remains the same in relative terms.

## 4. Discussion

Fundamental to the subsequent experimental investigation of the suitability of the marking agents, this work first focused on the systematic selection of a suitable marking agent for use in SLS. Using the selection methodology, the alternative of modified polymers was evaluated as the most suitable alternative, followed by the alternative of fluorescent particles. The reason for this result is that the modified polymers do not affect the particle shape and PSD, as well as the flowability and processability of the sinter material. In addition, modified polymers have a higher information content than fluorescent particles. The fluorescent particles have a lower analysis time than modified polymers. This criterion does not have a significant effect on the result due to the existing application in SLS for analysis on demand and the criteria weighting. Furthermore, the fluorescent particles have a higher thermal stability than modified polymers. However, this condition is not relevant for the result due to the selected preference function. Although the silicate-encapsulated DNA has a higher information content and a more spherical particle shape than the other alternatives investigated, the criteria of commercial availability and thermal stability, as well as their weighting, have a significant effect on the suitability of the alternative for the present application. Furthermore, the fluorescent particles and the silicate-encapsulated DNA have a different color expression than the modified polymers. However, due to its low weighting, the color criterion is not decisive for the present decision.

Only generally available information on the marking agents and information from manufacturers was used in the selection process. In this work, properties of the marking agents were evaluated that may not correspond to the actual production process. Validation of this work in terms of traceability of the materials and feasibility for the SLS process is still to be conducted. A more in-depth investigation of material properties may be useful in the context of decision support and will provide a better basis for comparison. Provided an enlarged data base as well as other potential approaches to material marking are available, other marking technologies should also be considered in the decision-making process, possibly resulting in a different outcome within the scope of this work.

Due to the available information on the investigated marking agents (Table 1), only the targets listed in Figure 3 were investigated. The hierarchical target system (Figure 3) is exclusively suitable for the investigation carried out in this work. The selection of the individual target groups and targets as well as the design of the criteria is crucial for the complete decision support process. Therefore, for further investigations based on the selection methodology, the correctness of the underlying hierarchical target system must always be verified. In addition, the used target system can be supplemented by further dimensions and the evaluation result should be validated by experimental investigations. For example, the influence on the mechanical component properties [71] and the effect of the marking agent on the haptics of the resulting components [2,83,84,85] can be taken into account. This validation did not take place within the scope of this work, but will be part of a subsequent work. The chemical compatibility of the marking agent with the sinter material is decisive for the influence on the mechanical component properties [71,72]. The media compatibility of the marking agent must also be taken into account. Although only very low concentrations of a marking technology are present in the sinter material and no significant changes in the material and component properties are to be expected as a result, the influence of the marking technology must nevertheless be checked in subsequent investigations. In addition, the methodical influencing variables in SLS mentioned in Wegner [86], such as the process parameters in the manufacturing process, must be taken into account. According to this, the marking agent must not lead to an adjustment of the process parameters [2,84,85,87]. Moreover, the criterion of material marking costs of the investigated alternatives was not considered, since these are both partly still in the research stage and commercially not or only limited available. The material marking costs could vary depending on the available amount of material to be marked due to economies of scale. Furthermore, the present result of the evaluation is only valid for the underlying sinter material specified, which can differ, among other things, in particle shape, PSD, and color characteristics (Table 2 and Figure 1).

The use of the considered alternatives of modified polymers [37,55,59,60,62,88] and silicate-encapsulated DNA [53,58] is associated with high investment costs, especially due to the analytical technique (Table 1). This evaluation criterion was not considered in the present work. Paunescu et al. [65] emphasize economies of scale by minimizing the use of marking agents in the sinter material, although the profitability of this approach must first be proven in a real application. In the context of this work, only tracing processes for continuous storage of information from the starting material to the component were considered. The use of in-process databases offers a proven, low-cost alternative in conjunction with tracking methods such as data matrix or quick response coding on powder reservoirs and components [89] to the tracing approach. This presents a challenge for a marker-based tracing methodology.

The applied methodology allows a structured construction of the hierarchical target system as well as an in-depth discussion for preference determination. Furthermore, the expression of indifferences and incomparabilities is possible. In essence, it has to be considered in the applied methodology that in such a multi-criteria decision support process, subjective individual decisions always influence the result. Subjectivity manifests itself, for example, in the weighting of individual criteria by pairwise comparison or in the ranking of alternatives based on preference functions. Thus, a biased, manifested basic attitude has a lasting influence on the outcome of the decision support process. The user must always be aware of this limitation and take possible countermeasures. It is conceivable to carry out the decision process in a plenum. In addition, there are a variety of other methods for supporting a decision process [45,47] than can be used for the present use case.

## 5. Conclusions and Outlook

The idea of a marker-based traceability methodology for the purpose of process optimization was presented. The use of a marking material in SLS provides an agile system for the rapid and simple detection of spreading material properties such as the aging state and the composition of a polymer powder. Based on the provision of valid process, equipment, and component data, in-process quality assurance and timely correction of process parameters for the purpose of compensating for the material properties at hand are enabled. Fundamental to the subsequent experimental investigation of the suitability of the marking agents as well as the development of the tracing strategy in part II of the study, the aim of this work was initially the systematic selection of a suitable marker for use in SLS, taking into account the sinter material to be marked. Therefore, a set of marking technologies was evaluated using the selection methodology. The main conclusions can be summarized as follows:

- The evaluated marking agents differ fundamentally in the functional principle, the number of possible codes, and the analysis method;
- The applied methodology for the systematic selection of marking agents, based on a combination of utility analysis and the PROMETHEE method, is characterized by a structured design of the target system, an in-depth analysis for preference determination, and the expression of indifferences and incompatibilities;
- The modified polymers are evaluated as the most suitable marking agent;
- This result is due to the information content, particle shape, and PSD, as well as the thermal stability and commercial availability of the modified polymers.

Although the investigations carried out show that the applied methodology can be used to support the decision-making process for selecting the most suitable marking agent for SLS, this work creates scope for further investigations. Regarding the applied methodology, a deeper comparison of the different marking technologies sparks interest in order to verify and possibly extend the developed methodology to other approaches. For example, the PROMETHEE method offers further possibilities for displaying the results by means of layer visualizations and evaluations based on a preorder [45]. The marking technologies under study need be further investigated in the context of SLS. The experimental validation of these results with regard to the traceability of the materials and the feasibility for the SLS process will be presented in the part ΙΙ of this study. In particular, the influence of the marking agent on the material and component properties as well as the effect of the SLS process on the stability of the marking agent are to be investigated in the future. Based on the used marking agent and the experimental findings, a suitable coding strategy is to be developed. For this purpose, specific concentrations and/or types of marking agents could be added to the sinter material, which can be uniquely identified at different, predefined stations in the SLS process chain. A promising approach for further investigations is the use of components as combined material data storage [59,90,91]. The storage of manufacturer-, process-, or product-related data in a product could be an alternative or complement to blockchain technologies [92].

## Figures and Tables

**Table 1:** Available properties of the investigated marking agents.

Criterion	Modified Polymers	References	Fluorescent Particles	References	Silicate-Encapsulated DNA	References
Thermal resistance	380 °C	[51,52]	800 °C–1700 °C	*	140 °C	[53]
Additional process	Yes	[51,52], Application by spray drying and mixing process	Yes	Mixing process	Yes	Mixing process
Particle shape	Analogous to sinter material	Application by spray drying	Jagged	*	Spherical	[53]
Particle size distribution	Analogous to sinter material	Application by spray drying	Customizable	*	60 nm–250 nm	[53]
Concentration in sinter material	1 ppm	[37,51,52,54,55]	>800 ppm	*, [56,57]	<1 ppm	[58]
Color deviation from sinter material	No	Analogous to sinter material, due to application by spray drying	Yes	*	Yes	[53]
Analysis duration	~1 min	[59,60]	10 ms–1000 ms; ~100 ms	*	90 min	[61]
Type of test	Non-destructive (DESI-MS/MS **)	[60,62]	Non-destructive (Fluorescence spectrometry)	[56]	Destructive (PCR ****-duplication and gel electrophoresis)	[53,58]
Harmlessness ***	Yes	[63]	Yes	[64]	Yes	[65]
Commercial availability	Yes	[37,51,52,62]	Yes	[64]	No	/
Number of possible codings	2^64^ (64 Bits)	[55]	2^10^ (10 Bits)	[56]	4^60^ (120 Bits)	[65]

* Manufacturer information. ** Desorption electrospray ionization tandem mass spectrometry. *** Harmlessness in the present concentration in the sinter material. **** Polymerase chain reaction.

**Table 2:** Investigated properties of the used sinter material.

Property	Expression
Melting temperature	181.9 °C
Sphericity	0.838
Aspect ratio	0.710
Particle size distribution	28.2 µm-d10 66.3 µm-d50 100.0 µm-d90

**Table 3:** Assessment points of the individual criteria.

Number	Criterion	Assessment Point
C1	Color embossing	Different color
C2	Additional process	Yes/No
C3	Particle shape	Spherical, “potato shaped”, jagged
C4	Particle size distribution	Between 28.2 µm-d10 and 100.0 µm-d90
C5	Analysis duration	Time [min]
C6	Non-destructive testing	Yes/No
C7	Harmlessness	Yes/No
C8	Commercial availability	Yes/No
C9	Information content	Number of codings
C10	Thermal resistance	At least 200 °C

**Table 4:** Pairwise comparison for the weighting of the criteria examined. The pairwise comparison is carried out by the authors. The weighting results from the ratio of the sum of the single criterion to the total sum of the criteria.

Criteria	C1	C2	C3	C4	C5	C6	C7	C8	C9	C10	Ʃ	Weight
**C1**	-	2	2	2	2	3	1	1	2	1	16	0.059
**C2**	4	-	2	2	4	4	1	2	4	1	24	0.089
**C3**	4	3	-	3	4	4	1	2	4	1	26	0.097
**C4**	4	4	3	-	4	4	1	2	4	1	27	0.100
**C5**	4	2	2	2	-	4	1	2	4	1	22	0.082
**C6**	3	2	2	2	2	-	1	1	3	1	17	0.063
**C7**	5	5	5	5	5	5	-	4	5	4	43	0.160
**C8**	5	4	4	4	4	5	2	-	4	2	34	0.126
**C9**	4	2	2	2	2	3	1	2	-	1	19	0.071
**C10**	5	5	5	5	5	5	2	4	5	-	41	0.152
											269	1.0

**Table 5:** Assignment of the used preference functions.

Number	Criteria	Preference Function	q	p
C1	Color embossing	Ordinary criterion	-	-
C2	Additional process	Ordinary criterion	-	-
C3	Particle shape	Criterion with linear preference and indifference range	Similar particles	Spherical versus fissured particles
C4	Particle size distribution	Criterion with linear preference and indifference range	[28.2 µm; 100.0 µm]	±30 µm outside the interval
C5	Analysis duration	Criterion with linear preference	-	>30 min difference
C6	Non-destructive testing	Ordinary criterion	-	-
C7	Harmlessness	Ordinary criterion	-	-
C8	Commercial availability	Ordinary criterion	-	-
C9	Information content	Criterion with linear preference	-	>100 Codes difference
C10	Thermal resistance	Ordinary criterion	-	-

**Table 6:** PROMETHEE matrix with outranking relations and input and output flows [43,45,47].

	**a_1_**	**a_2_**	**a_3_**	ϕ^+^
**a_1_**	-	π_1,2_	π_1,3_	ϕ_1_^+^
**a_2_**	π_2,1_	-	π_2,3_	ϕ_2_^+^
**a_3_**	π_3,1_	π_3,2_	-	ϕ_3_^+^
ϕ^−^	ϕ_1_^−^	ϕ_2_^−^	ϕ_3_^−^	

**Table 7:** Matrix of the determined outranking relations as well as input and output flows.

	a_1_	a_2_	a_3_	
**a_1_**	-	0.209	0.584	0.793
**a_2_**	0.082	-	0.524	0.606
**a_3_**	0.167	0.167	-	0.335
	0.249	0.376	1.108	

**Table 8:** Ranking of the studied alternatives.

Placement	Alternative	Net Flow
1	Modified polymers (a_1_)	0.544
2	Fluorescent particles (a_2_)	0.230
3	Silicate-encapsulated DNA (a_3_)	−0.773

**Table 9:** Pairwise comparison as sensitivity analysis according to the “rank reversal” approach. The weighting and placement of the remaining criteria in relation to each other remains the same in relative terms.

Criteria	C2	C3	C4	C5	C6	C8	C9	C10	Ʃ	Weight	Old Placement	New Placement
**C2**	-	2	2	4	4	2	4	1	19	0.114	6	5
**C3**	3	-	3	4	4	2	4	1	21	0.126	5	4
**C4**	4	3	-	4	4	2	4	4	22	0.132	4	3
**C5**	2	2	2	-	4	2	4	1	17	0.102	7	6
**C6**	2	2	2	2	-	1	3	1	13	0.078	9	8
**C8**	4	4	4	4	5	-	4	2	27	0.162	3	2
**C9**	2	2	2	2	3	2	-	1	14	0.084	8	7
**C10**	5	5	5	5	5	4	5	-	34	0.204	2	1
									167	1.0		

## Data Availability

The raw/processed data required to reproduce these findings cannot be shared at this time as the data also form part of an ongoing study.
